# Management of Grade Three Open Distal Humerus Intra-articular Fracture Using the BB Joshi External Fixator: A Case Study and Review of Outcomes

**DOI:** 10.7759/cureus.66460

**Published:** 2024-08-08

**Authors:** Mukesh O Phalak, Archit Gupta, Swaroop Solunke, Abhishek Nair

**Affiliations:** 1 Orthopaedics, Dr. D. Y. Patil Medical College, Hospital and Research Centre, Dr. D. Y. Patil Vidyapeeth (Deemed to be University), Pune, IND

**Keywords:** elbow trauma management, distal humerus fracture, grade 3 open fracture, intra-articular fracture, bb joshi external fixator

## Abstract

Distal humeral intra-articular fractures often result in functional impairment if treated conservatively. These fractures are particularly challenging due to osteoporosis and severe comminution. A 32-year-old female with a grade three open distal humerus intra-articular fracture presented with a 5 cm x 5 cm open wound on her right elbow. The patient underwent open reduction and internal fixation (ORIF) using the BB Joshi external fixator and a local flap for skin coverage, achieving stable fixation and early mobilization. The patient regained a near-normal range of movements and adequate skin coverage of the wound at follow-up. Compared to traditional ORIF, it showed lower infection rates and comparable union rates, leading to better functional outcomes when compared with the studies reported earlier. The BB Joshi external fixator effectively treats grade three open distal humerus intra-articular fractures, minimizing complications and promoting functional recovery.

## Introduction

Fractures that occur within the joint of the lower end of the upper arm bone represent a small percentage (0.5-7%) of total fractures, but they make up a significant portion (30%) of fractures, specifically in the elbow [1]. Distal humeral fracture usually occurs in younger individuals as a result of high-energy trauma, but in elderly women, it is more commonly caused by relatively low-energy trauma [1]. The likelihood of experiencing functional impairment and deformity is significantly elevated when using conservative treatment for distal intra-articular fractures of the humerus. Because of the complicated nature of the fracture and the existence of osteoporosis, achieving stable internal fixation may be difficult [2]. Good results can be achieved with ideal anatomical alignment, efficient stabilization, and rapid mobilization. The presence of bone loss, osteopenia, and severe comminution increases the likelihood of unsatisfactory outcomes due to insufficient stabilization of the fracture [2,3]. After therapy, approximately 25% of patients with these fractures face major difficulties, and a small percentage of patients might need another surgery [4]. Numerous surgical techniques, such as the use of reconstruction plates, K-wires, dynamic compression plates, screws, and locking compression plates, are used to repair both columns [5]. Here, we present a case of a grade three open distal humerus intra-articular fracture treated with a BB Joshi external fixator for stable fixation and a local flap for adequate skin coverage with a full post-operative range of movements.

## Case presentation

**Figure 1:**
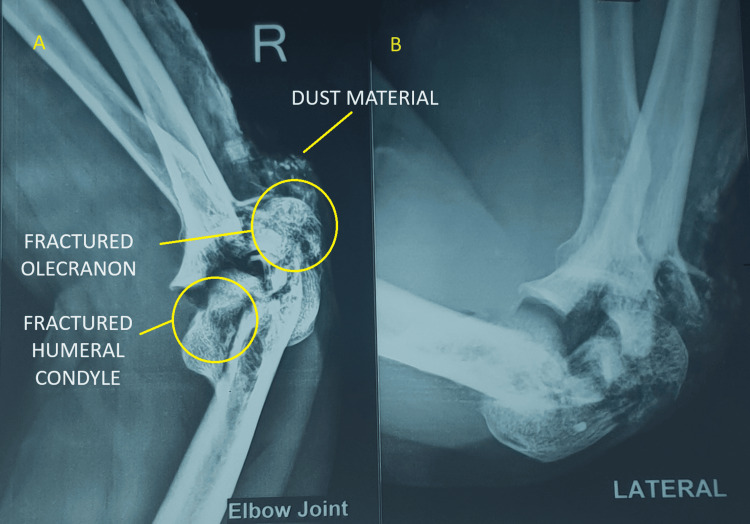
Pre-operative radiograph with anteroposterior (A) and lateral (B) view

A 32-year-old female patient presented to us with an open wound on the dorsum of her right elbow, measuring 5 cm × 5 cm, and reported an inability to move her elbow since a fall one day ago. There was no history of other injuries, significant medical conditions, or previous surgeries. On examination, her vital signs were stable. The local examination revealed a bone-deep open wound on the dorsum of the right elbow. Palpation identified a fracture fragment of the distal humerus and a partially avulsed triceps tendon from the olecranon, with a palpable fractured olecranon fragment within the wound. The assessment did not include an evaluation of the extent of movement, and no impairment was observed in the nerves or blood vessels further away from the injury site. A pre-operative radiograph was obtained, as shown in Figure 1A-1B.

Surgical procedure

**Figure 2:**
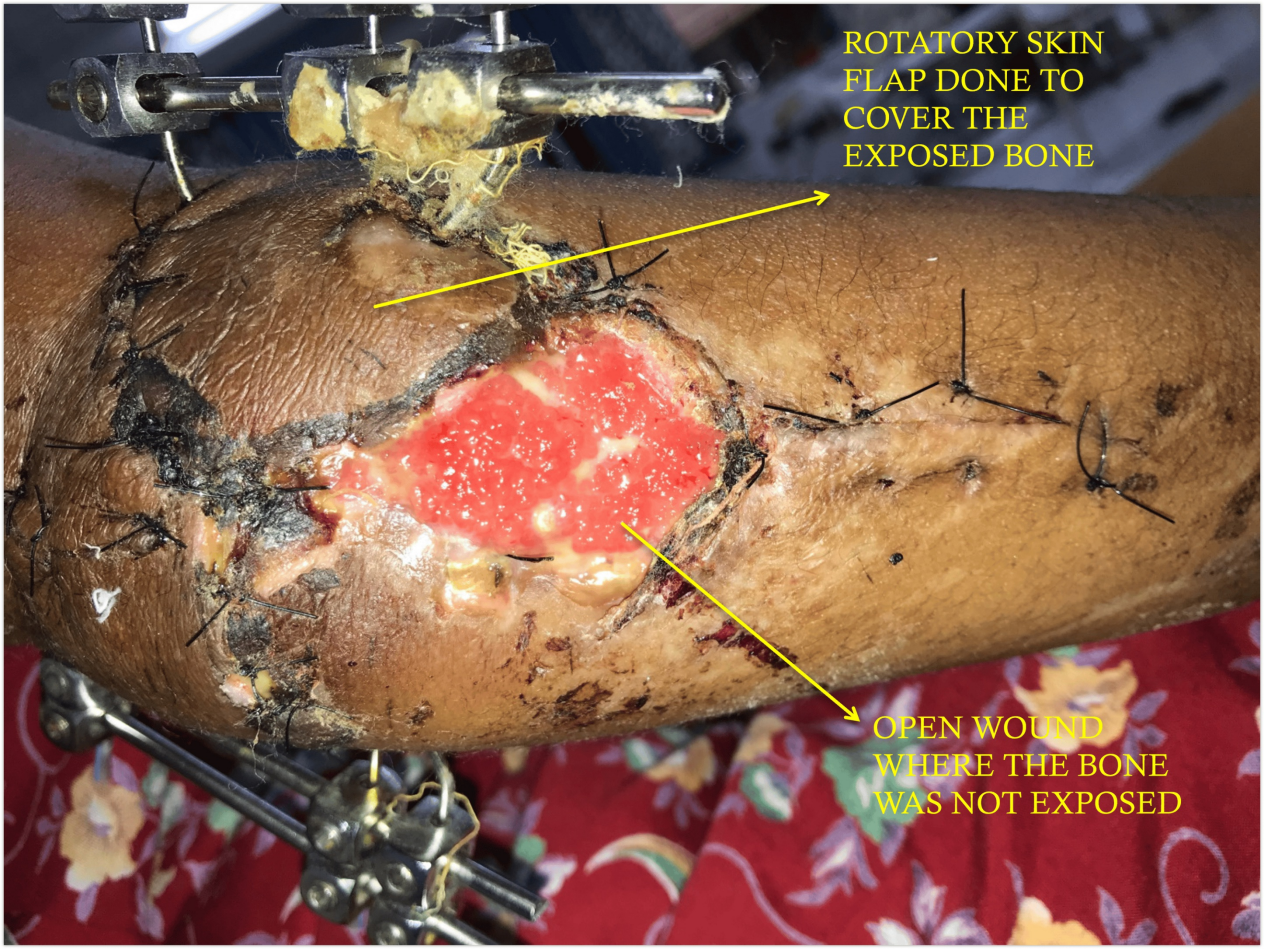
Immediate post-operative clinical photo with an external fixator in situ and a rotatory skin flap

**Figure 3:**
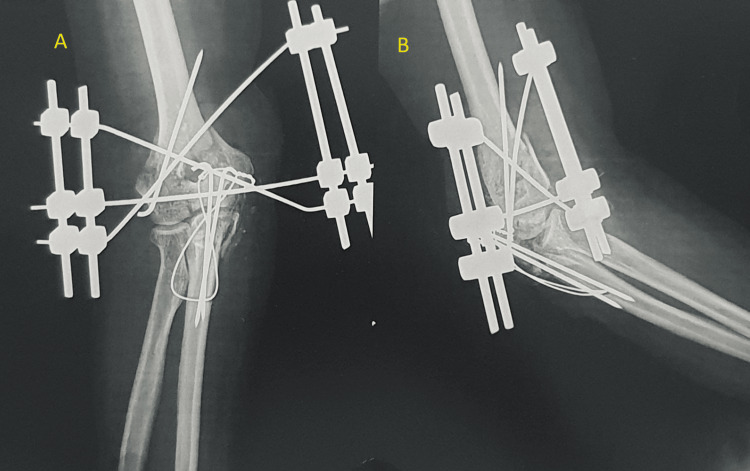
Post-operative radiograph of the right elbow with an external fixator in situ: (A) anteroposterior view and (B) lateral view

The surgical procedure involved open reduction and fixation with the BB Joshi external fixation. The patient was given anesthesia via a supraclavicular block and positioned laterally. After scrubbing, painting, and draping, a 3 cm incision was made over the right elbow, both above and below the open wound. The lateral and medial humeral condyles were realigned, and a 3 mm K-wire was inserted to maintain the alignment. Two K-wires had been inserted diagonally from the medial as well as lateral condyles and removed through the opposite side. A vertical fracture in the coronoid process was realigned and stitched with Vicryl No. 1. The olecranon was aligned and secured using K-wire tension band wiring. All Kirschner wires were connected externally with a clamp, and the triceps tendon was sutured with non-absorbable, braided polyester (Ethibond 2). A skin advancement flap was performed to cover the bone and hardware (Figure 2). After suturing the flap, a 2 cm x 2 cm defect remained on the posteromedial side of the elbow. All fragments of the olecranon and capitulum were held in anatomical position, and an external fixator was placed with three pins from the medial aspect and three from the lateral aspect, secured with clamps. The reduction was checked and found satisfactory under the C-arm. Closure was completed with Ethilon and Vicryl, followed by an aseptic dressing. The post-operative radiograph showed satisfactory reduction (Figure 3A-3B).

Rehabilitation

**Figure 4:**
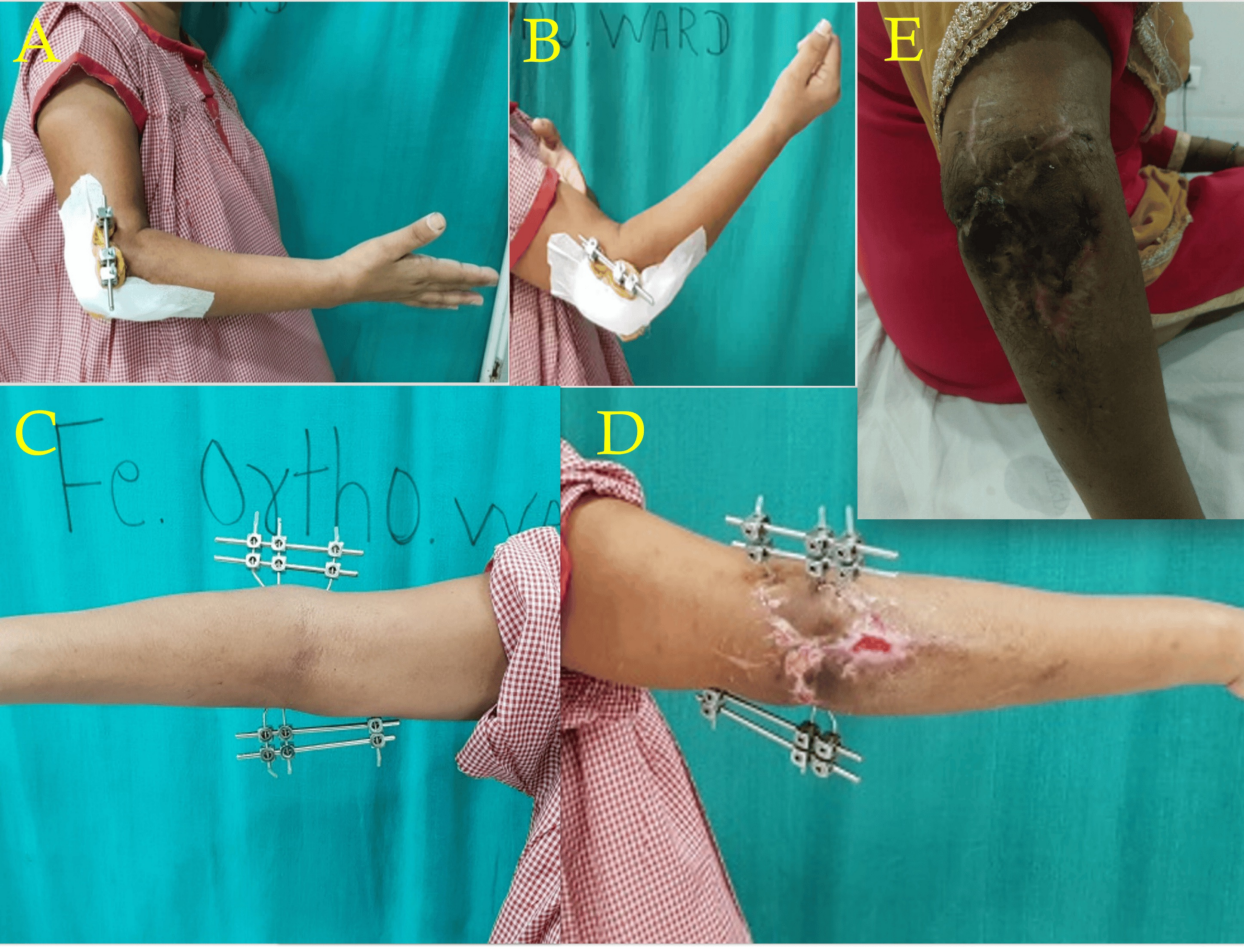
Post-operative rehabilitation with range of motion at the right elbow: (A) elbow extension at post-operative day 2, (B) elbow flexion at post-operative day 2, (C) volar aspect of the right elbow at the time of discharge on post-operative day 14, (D) dorsal aspect of the right elbow at the time of discharge on post-operative day 14, and (E) skin condition at three-month follow-up

The patient was started on a range of movement exercises on post-operative day 2. The sutures were removed on day 14, and the patient was discharged. At six weeks, the external fixator was removed. At the three-month follow-up, the wound had healed completely and the patient had a near-normal range of motion at the elbow joint. The clinical pictures of rehabilitation are shown in Figure 4A-4E.

Four-year follow-up

**Figure 5:**
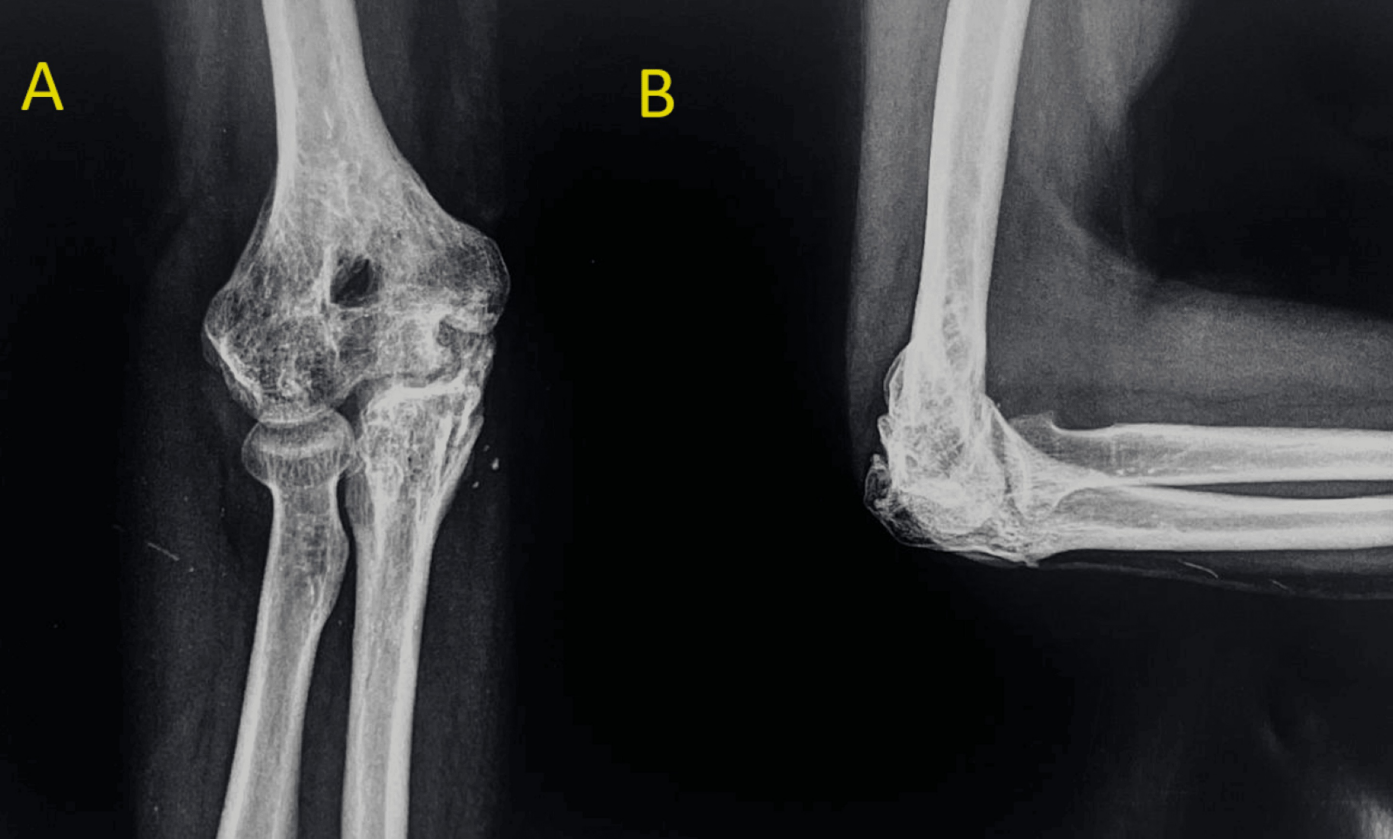
Follow-up radiograph after four years: (A) anteroposterior view and (B) lateral view

**Figure 6:**
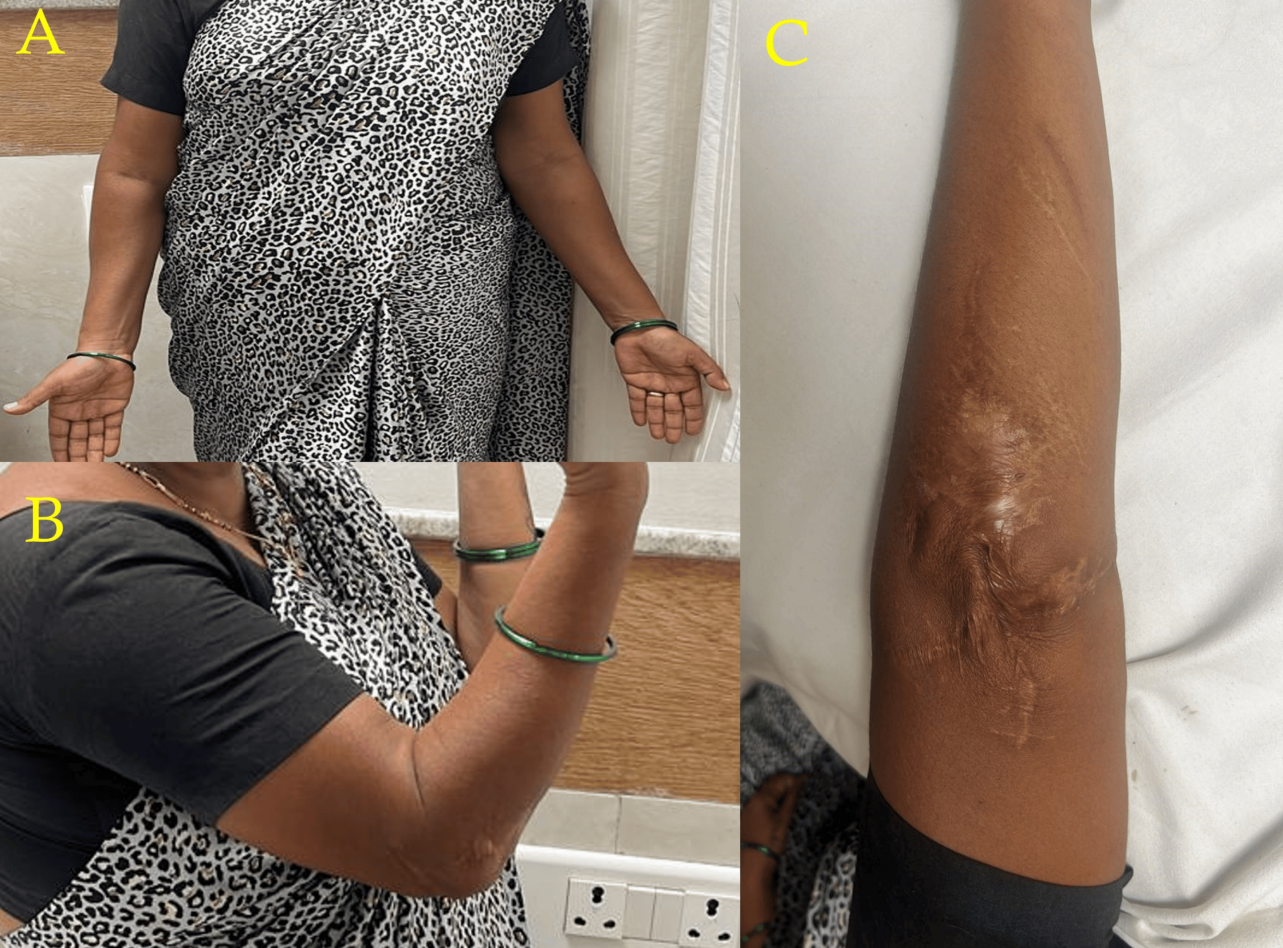
Range of motion at four-year follow-up: (A) anterior view of the bilateral elbows with full extension, (B) flexion at the right elbow, and (C) dorsal aspect of the right elbow

After four years of being operated on, the patient had no functional impairment, and the radiograph is shown in Figure 5. Figure 6 shows the range of movements and the wound after four years.

## Discussion

The management of grade three open distal humerus fractures presents a significant issue because of the severe nature of the injury, which often involves extensive soft tissue damage, contamination, and a high risk of infection. The four goals of operative treatment are to restore the diaphyseal bone stock, prioritize soft tissue healing without infection, achieve the union of the distal parts and shaft, and achieve stable and flexible articulation [6]. Using an external fixator like the BB Joshi is one of the most efficient ways to treat such complicated fractures.

As per the Gustilo-Anderson classification, grade three open fractures are distinguished by a greater possibility of vascular injury, significant soft tissue damage, and contamination [7]. These injuries are frequently the consequence of high-energy trauma, and to avoid consequences like infection, non-union, and malunion, careful debridement, stabilization, and soft tissue management are necessary. Fixing the fracture using the BB Joshi external fixator is an important component of the process. This device provides strong external stability, which is particularly beneficial in open fractures with significant soft tissue damage. Applying the BB Joshi fixator involves placing several pins on the medial as well as on the lateral sides of the humerus, which are subsequently joined externally using clamps to create a sturdy structure. The use of the BB Joshi external fixator allows for early mobilization, which is vital in preventing joint stiffness and promoting functional recovery [8].

Rehabilitation involves a structured physical therapy program focusing on gradual range of motion exercises along with strengthening of the surrounding musculature. Open reduction and internal fixation (ORIF) with plates and screws is the standard treatment for grade three open distal humerus fractures. Research by Ali et al. claims that ORIF enables early mobilization and stable fixation, both of which are essential for preserving joint function [9]. However, there are drawbacks to this approach, particularly when addressing serious soft tissue damage, as there is a much-increased chance of infection and non-union [10]. The literature indicates that one of the major complications associated with ORIF in grade three open fractures is infection, with rates reported as high as 27% in a few series [11]. Non-union and malunion are also notable concerns, with non-union rates ranging from 10% to 20% [12]. The extensive soft tissue damage in grade three fractures complicates the surgical approach and post-operative care, often necessitating additional procedures such as flap coverage or bone grafting [7].

The BB Joshi external fixator addresses some of these challenges by providing stable external fixation, which minimizes the need for extensive internal hardware that can exacerbate soft tissue damage and increase infection risk. A study by Sen et al. demonstrated that external fixation is particularly advantageous in managing open fractures with severe soft tissue involvement, as it allows for better management of the wound environment and reduces the likelihood of deep infection [13].

Comparison of outcomes with literature reports

Infection Rates

The BB Joshi external fixator has shown lower infection rates compared to traditional ORIF. In a comparative study, the infection rate in patients treated with external fixators was significantly lower, around 15%, compared to 27% with ORIF [13].

Healing and Union Rates

The healing and union rates are comparable between the two methods, with studies reporting satisfactory union rates of around 80% to 90% for both techniques [10,13]. However, the external fixator has the added benefit of reducing soft tissue complications, which can facilitate faster rehabilitation.

Functional Outcomes

Functional outcomes, as measured by the range of motion and patient-reported outcomes, are also favorable with the BB Joshi external fixator. According to research by Gupta and Sunil, patients who received external fixators had higher elbow function scores than those who had internal fixation [14]. This is attributed to the ability to initiate early joint mobilization, which is crucial for preventing stiffness and maintaining joint function.

Rehabilitation and Long-Term Results

Rehabilitating patients with distal humerus fractures is essential. The BB Joshi external fixator allows for early mobilization, which is consistent with the literature emphasizing the importance of early motion in achieving optimal functional outcomes [8]. Long-term results from studies on external fixation indicate sustained functional improvement and patient satisfaction, with fewer secondary interventions required compared to ORIF [12].

## Conclusions

The management of grade three open distal humerus fractures remains a formidable challenge due to the extensive soft tissue damage, contamination, and high risk of infection associated with these injuries. The primary goals of operative treatment are to restore bone stock, ensure soft tissue healing, achieve union of the distal parts and shaft, and maintain stable and flexible articulation. The BB Joshi external fixator has emerged as a highly effective treatment modality for these complex fractures. By providing robust external stability and minimizing the need for extensive internal hardware, the BB Joshi fixator helps reduce infection rates and supports early mobilization, which is crucial for preventing joint stiffness and promoting functional recovery. Although ORIF with plates and screws remains a standard treatment, it carries significant risks, particularly regarding infection and non-union, especially in the presence of severe soft tissue damage. Comparative studies indicate that the BB Joshi external fixator offers lower infection rates, comparable healing and union rates, and favorable functional outcomes. Early mobilization, facilitated by the external fixator, contributes to better long-term results and patient satisfaction. Overall, the BB Joshi external fixator presents a compelling alternative to traditional ORIF, particularly in managing grade three open distal humerus fractures with substantial soft tissue involvement.
